# Parent experiences questionnaire for outpatient child and adolescent mental health services (PEQ-CAMHS Outpatients): reliability and validity following a national survey

**DOI:** 10.1186/1753-2000-5-18

**Published:** 2011-05-21

**Authors:** Andrew M Garratt, Oyvind A Bjertnaes, Olaf Holmboe, Ketil Hanssen-Bauer

**Affiliations:** 1 Norwegian Knowledge Centre for the Health Services, Oslo, Norway; 2 Centre for Child and Adolescent Mental Health, Eastern and Southern Norway, Oslo, Norway; 3 Department of Research and Development, Division of Mental Health Services, Akershus University Hospital, Lørenskog, Norway

## Abstract

**Abstact:**

**Background**

Development and evaluation of the PEQ-CAMHS Outpatients, a parent completed questionnaire to measure experiences of outpatient child and adolescent mental health services (CAMHS) in Norway.

**Methods**

Literature review, parent interviews, pre-testing and a national survey of 17,080 parents of children who received care at one of the 86 outpatient CAMHS in Norway in 2006. Telephone interviews were conducted with a random sample of non-respondents. Levels of missing data, factor structure, internal consistency and construct validity were assessed.

**Results**

7,906 (46.0%) parents or primary caregivers responded to the questionnaire. Low levels of missing data suggest that the PEQ-CAMHS is acceptable. The questionnaire includes three scales supported by the results of factor analysis: relationship with health personnel (8 items), information and participation (4 items), and outcome (3 items). Item-total correlations were all above 0.6 and Cronbach's alpha correlations ranged from 0.88-0.94. The results of comparisons of scale scores with several variables relating to global satisfaction, outcome, cooperation, information, involvement and waiting time support the construct validity of the instrument.

**Conclusions**

The PEQ-CAMHS Outpatients questionnaire includes important aspects of outpatient CAMHS from the perspective of the parent. It has evidence for data quality, internal consistency and validity and is recommended in surveys of parent experiences of these services. Future research should assess test-retest reliability and further tests of construct validity that include clinical data are recommended.

## Background

The widespread acceptance of the importance of the views of service users in the assessment of health care quality has meant that the views of parents are increasingly sought in evaluations of the quality of care of child and adolescent mental health services (CAMHS) [1,2]. Compared with the patient satisfaction literature more generally, [3] there have been relatively few studies of user experiences with CAHMS but there is some evidence for small to moderate levels of correlation between satisfaction and mental health outcomes for children [4-7].

Parents have responsibility for contacting health services on behalf of their child and their involvement in treatment is important for its success [8-10]. Moreover, parent experiences of care might influence their expectations of and involvement in future care [1,11]. Hence, parents can make an important contribution in the evaluation of mental health services quality for children and parent-completed questionnaires have had applications across a wide range of evaluations that include medication [12,13], community-based services [1,14,15], inpatient care [7,11] and health care plans [16].

Reviews of the literature have identified up to 28 instruments and questionnaires designed to measure parent satisfaction with CAMHS [9,17]. These questionnaires typically comprise multidimensional scales that assess different aspects of parent satisfaction or experiences with such services. However, some questionnaires comprise global items while others sum to form a single score that might assess one aspect of parent satisfaction or general satisfaction [18]. Global items have been criticised for not providing adequately detailed information [9,11,19]. Multidimensional instruments provide more detailed information relating to specific aspects of care such as communication and information that can more fully inform quality improvement initiatives.

Parent satisfaction questionnaires have been criticised more generally for not being comprehensive [20] and for lacking psychometric evidence including reliability and validity[7,9,20]. The former criticism relates to content validity, a qualitative consideration as to whether an instrument or questionnaire adequately covers important aspects of parent satisfaction and in sufficient depth. An alternative approach involves asking users to rate their experiences of aspects of health care which may include communication and information provision, [21] and the collection of more objective information relating to whether specific health care events occurred. There is an inbuilt assumption that the aspects of experience covered by such questionnaires are related to user satisfaction and tests of construct validity often involve comparisons with global or single item measures of satisfaction [21]. It is important that users are involved in the development of such questionnaires to ensure that the most relevant aspects of the health care experience are included which lends the questionnaire content validity.

The Parent Experiences Questionnaire for Outpatient Child and Adolescent Mental Health Services (PEQ-CAMHS Outpatients) was developed following a review of the literature that identified shortcomings with existing questionnaires including a lack of content relevance to outpatient clinics in Norway. The questionnaire was designed for application in a national survey of parents or primary caregivers with children under 16 years of age attending mental health services outpatient centres throughout Norway [22]. The aim of the present study was to assess the data quality, internal consistency reliability and validity of the questionnaire.

## Methods

### Questionnaire development

Questionnaire development and evaluation followed criteria that have been defined as important in patient satisfaction measurement [23]. The development of the questionnaire was based on a review of the literature that included existing parent satisfaction questionnaires, interviews with parents and consultation with an expert group comprising nine health professionals, a representative from a user organisation and researchers. This process was designed to ensure content validity; that the questionnaire addresses important aspects of parent experiences in sufficient detail.

Semi-structured interviews were undertaken with parents of eleven children under 16 years of age that had received care from two outpatient CAMHS in South East Norway. Following the interviews and any necessary revisions, the expert group was consulted. The questionnaire was then piloted by means of a postal questionnaire sent to 271 parents of children under 16 years of age that had attended one outpatient CAMHS in South East Norway. The expert group was consulted again following the pilot survey.

### Data collection

The questionnaire was included in national survey of 17,871 parents of children aged under 16 years who had at least one appointment at one of the 86 outpatient centres in Norway between 1 September and 31 December 2006 [22]. Patients were recruited using administrative data from the clinics and there was a maximum of 400 users per clinic who were randomly selected if the number exceeded 400 during the recruitment period. This calculation was based on the number of respondents needed to compare the 86 clinics after taking account of non-response and the need for adjustment of covariates.

Parents were sent a postal questionnaire following the transfer of administrative data from the clinics. The covering letter informed them that the survey related to parent experiences. No instruction was given about which parent should complete the questionnaire and a question was included about who actually responded. Parents gave their informed consent to take part by ticking a box on the front page of the questionnaire which was returned in a reply paid envelope. Up to two reminders were sent to non-respondents at three and six weeks.

Telephone interviews were conducted with a sample of 400 non-respondents following the second reminder; 20 centres were randomly selected from the 86 centres in Norway and 25 patients were randomly selected for each centre. The interviews included four items that were found to explain the largest component of variation in parent experiences following the pilot survey along with background questions. These four questions were summed to form a summary score which had satisfactory item-total correlations and a Cronbach's alpha of 0.84. The two groups of parents were compared using the t-test for the summary score and chi-square and Mann-Whitney U tests for categorical variables and ordinal variables.

The Norwegian Regional Committee for Medical Research Ethics, the Data Inspectorate and the Norwegian Board of Health approved the survey.

### Statistical analysis

Items with ten percent or more missing data were considered for removal from the final questionnaire. Principal axis factoring with promax rotation was used to assess the underlying structure of the questionnaire [24]. Factors with eigenvalues above 1.0 were assessed for face validity and factor loadings greater than 0.4 were considered important. Items with important factor loadings on more than one factor were considered for removal. Internal consistency was assessed by item-total correlation and Cronbach's alpha. The former measures the strength of association between an item and the remainder of its scale and it was expected that they would exceed 0.4 which is considered acceptable [25]. The latter assesses the overall correlation between items within a scale. For a scale to be considered sufficiently reliable for use in groups of patients, an alpha value of 0.7 is considered acceptable [25,26].

In the absence of a gold standard measure of parent experiences, construct validity was assessed through comparisons of scale scores with responses to the additional questionnaire items not included in the scales that related to various aspects of care. Following findings from the parent satisfaction literature, [27,28] it was hypothesised that moderate to high levels of correlation in the range 0.5 to 0.7, would be found between scale scores and parents overall satisfaction both in relation to their child's treatment and their own experiences. There is some evidence for a low to moderate association between parent satisfaction and treatment outcomes for mental health services [4-7], and correlations in the range 0.3 to 0.6 were hypothesised for the perceived benefits of treatment accruing to the child and parent. Similar levels of correlation were hypothesised for responses to two items relating to health professionals cooperation, both within the clinic and with other professionals working outside the clinic including general practitioners and school teachers.

The active participation of parents and families in mental health care of children and adolescents leads to better outcomes including parent satisfaction [10,14] and parent satisfaction was found to have low to moderate correlations with a measure of empowerment [10]. Hence, levels of correlation in the range 0.3 to 0.6 were expected between the scales and two items relating to parent understanding of treatment and involvement in dialogue with the clinic. Information giving is also positively related to user satisfaction with health services [3] and it was hypothesised that scale scores would have low to moderate correlations in the range 0.3 to 0.6 with perceptions of the adequacy of information relating to side-effects of their child's medication.

There is evidence that parents are more satisfied with shorter waiting times [14,28] and it was hypothesised that scores would have low levels of correlation in the range 0.2 to 0.3 with how long parents had to wait for treatment for their children. Parent satisfaction ratings have been found to be positively associated with the time spent in treatment with mental health services [19,29,30] and hence it was hypothesised that scores would have low to moderate correlations in the range 0.3 to 0.6 with parent perceptions of the adequacy of the number of hours spent at the clinic, number of clinic visits and the ease with which they were able to make contact with health professionals outside of consultation times when necessary.

Unwanted behaviour by health professionals has been found to be consistently associated with user satisfaction more generally [3] and following previous findings from user satisfaction with mental health services [31], low correlations in the range 0.2 to 0.3 were expected with responses to two items relating to whether health professionals talked down to either the child or their parent.

## Results

### Questionnaire development

Following the literature review and consultation with the expert group 28 questions were devised that covered accessibility, cooperation, information, overall satisfaction, outcomes and relationship with health personnel. These questions were included in the semi-structured interviews with the parents of eleven children. In general the questions were found to be acceptable and relevant. Some revisions were made following the interviews and consultation with the expert group.

Of the 271 parents sent the pilot questionnaire, 122 (45.0%) responded. The mean age of respondents was 40.83 (sd 6.67) and 93 (76.2%) were the mother. Items had low levels of missing data and factor analysis of 14 items that were relevant to all patients identified three scales of relationship with health personnel, information and participation and outcome. Following consultation with the expert group some small changes were made to the wording of some of the items prior to the main survey and an additional item relating to "how the child functions outside of the family" was included as part of the outcome scale. Hence the PEQ-CAMHS Outpatients questionnaire included 15 items assessing parent experiences with five-point descriptive scales that assess various aspects of parent experiences of relevance to all parents.

Additional items relating to information on medication information and cooperation between health professionals that were not relevant to all parents' included a "not applicable" option and other additional single items covered accessibility, overall outcome, overall satisfaction and unwanted behaviour. Finally, the questionnaire included several sociodemographic questions.

### Data collection

Of the 17,871 parents mailed the national survey questionnaire, 7,906 responded; 755 did not have a correct address and 36 had cancelled their CAMHS appointment which gives an adjusted response rate of 46.3%. Table 1 shows their characteristics. The mean age of the children was 11.3 (SD = 3.2) years and 2,946 (37.3%) were female. Of the 400 non-respondents conducted by telephone, 225 (56.3%) agreed to be interviewed. Compared to respondents to the main survey, there were three statistically significant differences that were important. Interviewees had a significantly different number of previous contacts with the clinic with 21.0% of interviewees and 14.4% of respondents reporting more than one previous contact. Interviewees also had a significantly different education level with 25.3% having received higher education compared with 44.3% for respondents. The parent experiences summary scores were not significantly different. However, interviewees had slightly poorer experiences relating to their "viewpoints being taken seriously". There was also a significant difference for the number of clinic visits in the past three months. There were a large number of interviewees that had not visited the outpatient clinic in the past three months which follows from the interviews taking place after the second reminder.

**Table 1:** Child and parent characteristics for the main survey (n = 7,906) and non-respondents who were telephoned^a ^(n = 225)

Variable	Main survey N (%)	Telephone interviews N (%)
Sex of patient		
Female	2946 (37.3)	82 (36.4)
Male	4947(62.7)	143 (63.6)
Mean (sd) age of patient	11.32 (3.22)	11.15 (3.39)
Sex of parent completing questionnaire		
Female	6515 (85.3)	173 (84.4)
Male	1124 (14.7)	34 (15.6)
Mean (sd) age of parent	40.38 (6.87)	39.88 (7.13)
Education level of parent		
Primary school	827 (10.8)	45 (20.4)**
High school	3456 (44.9)	120 (54.3)
University graduate	2049 (26.6)	39 (17.6)
University postgraduate	1361 (17.7)	17 (7.7)
First language of parent		
Norwegian	7292 (94.7)	214 (96.0)
Sami	20 (0.3)	2 (0.9)
Other Scandinavian	87 (1.1)	3 (1.3)
Other European	150 (1.9)	0
Not European	149 (1.9)	4 (1.8)
Main activity of parent in the past week		
Work	5203 (69.8)	119 (55.3)
Sick leave	1322 (17.7)	61 (28.4)
Education	332 (4.5)	9 (4.2)
Home worker	453 (6.1)	20 (9.3)
Unemployed	149 (2.0)	6 (2.8)
No. previous contacts with clinic		
0	5674 (74.2)	156 (72.9)**
1	877 (11.5)	13 (6.1)
>1	1100 (14.4)	45 (21.0)
No. clinic visits in past three months		
0	0	71 (32.7)**
1	2520 (35.2)	37 (17.1)
2-5	3255 (45.5)	73 (33.6)
6-12	1224 (17.1)	29 (13.4)
12-	153 (2.1)	7 (3.2)
Mean (sd) parent clinic knowledge	3.79 (1.04)	3.76 (1.03)
Very poor	321 (4.3)	5 (2.3)
Fairly poor	513 (6.8)	18 (8.4)
Neither poor nor good	1582 (21.0)	62 (28.8)
Fairly good	3141 (41.7)	69 (32.1)
Very good	1968 (26.2)	61 (28.4)
Child badly treated?		
No	7310 (95.6)	218 (99.1)
Once	188 (2.5)	1 (0.5)
Sometimes	128 (1.7)	1 (0.5)
Many times	23 (0.3)	0
Mean (sd) parent experiences scores^b^	65.29 (21.13)	64.10 (21.10)
Care and consideration - parent^c^	2.81 (0.93)	2.71 (0.94)
Took viewpoints seriously	3.04 (0.91)	2.91 (0.86)**
Information - treatment	2.11 (1.12)	2.19 (1.12)
Overall outcome - parent	2.46 (1.09)	2.51 (1.09)

^a^T-test - age, parent experiences scores; Mann-Whitney test - parent experiences items, parent clinic knowledge; Chi-Square test - sex, education level, first language, main activity, number of previous contacts, number of clinic visits, child badly treated.

Asterisks denote statistically significant differences between the two groups: *p < 0.05;**p < 0.01.

^b^Sum of the four items transformed to 0-100 where 100 is the best possible experiences of care.

^c^Items are scored from 0-4 where 4 is the best possible experiences of care.

### Statistical analysis

The levels of missing data and descriptive statistics are shown in Table 2. All items met the criterion for missing data and none were removed. Missing data ranged from 1.5% to 5.6% for items relating to "being met with politeness and respect" and "outcomes associated child's function outside of the family" respectively. These two items also had the highest and lowest scores of 4.35 and 2.39 respectively. The majority of items had mean scores in the range of 3 to 4 on the scale of 1 to 5 where 5 is the best possible experiences of care.

**Table 2:** Descriptive statistics, factor analysis and internal consistency

Scale/item	Missing (%)	Mean (sd)	Frequency (%)					Factor loading	Cronbach's alpha (*scale*)/item-total correlation
			1	2	3	4	5^a^		
*Relationship with health personnel*^b^	172 (2.2)	74.92 (18.43)^c^							0.94
Care and consideration - parent	170 (2.2)	3.81 (0.93)	148 (1.9)	501 (6.5)	1813 (23.5)	3438 (44.6)	1800 (23.4)	0.82	0.82
Understanding of situation	256 (3.3)	3.85 (0.94)	162 (2.1)	45 (5.9)	1701 (22.3)	3372 (44.3)	1927 (25.3)	0.84	0.84
Care and consideration - child	273 (3.5)	4.14 (0.82)	49 (0.6)	216 (2.8)	1110 (14.6)	3431 (45.2)	2791 (36.7)	0.82	0.78
Met with politeness and respect	116 (1.5)	4.35 (0.73)	49 (0.6)	90 (1.2)	616 (7.9)	3359 (43.3)	3640 (46.9)	0.91	0.78
Spoke in a way that was understandable	242 (3.1)	4.33 (0.71)	30 (0.4)	97 (1.3)	585 (7.7)	3529 (46.3)	3387 (44.4)	0.74	0.66
Took viewpoints seriously	274 (3.5)	4.04 (0.91)	125 (1.6)	321 (4.2)	1275 (16.8)	3251 (42.8)	2624 (33.3)	0.82	0.83
Had enough time for contact/conversation	258 (3.3)	3.90 (0.97)	261 (3.4)	977 (12.8)	2122 (27.9)	2865 (37.6)	1387 (18.2)	0.54	0.69
Cooperation with parent	194 (2.5)	3.54 (1.04)	180 (2.3)	489 (6.4)	1457 (19.0)	3333 (43.4)	2217 (28.9)	0.76	0.86
*Information and participation*^b^	273 (3.5)	59.56 (23.72)							0.88
Participation in choice of treatment	323 (4.1)	3.59 (1.08)	394 (5.2)	776 (10.3)	1921 (25.5)	2911 (38.6)	1545 (20.5)	0.89	0.79
Inflluence in choice of treatment	392 (5.0)	3.39 (1.22)	491 (6.6)	1058 (14.1)	2225 (29.8)	2492 (33.3)	1212 (16.2)	0.91	0.76
Information - treatment	376 (4.8)	3.11 (1.12)	668 (8.9)	1502 (20.0)	2478 (33.1)	2002 (26.7)	844 (11.3)	0.75	0.75
Information - child's condition	365 (4.6)	3.43 (1.10)	449 (6.0)	1018 (13.6)	2117 (28.2)	2631 (35.1)	1290 (17.2)	0.53	0.65
*Outcome*^d^	367 (4.6)	72.08 (21.32)							0.91
Child's outcome from treatment	324 (4.1)	3.96 (0.94)	110 (1.5)	202 (2.7)	2187 (29.0)	2394 (31.7)	2653 (35.2)	0.90	0.84
Child's function in the family	382 (4.9)	3.81 (0.93)	107 (1.4)	175 (2.2)	2574 (32.7)	2366 (30.1)	2266 (28.8)	0.89	0.86
Child's function outside of family	441 (5.6)	2.39 (0.92)	128 (1.7)	239 (3.2)	2557 (34.4)	2469 (33.2)	2036 (27.4)	0.83	0.89

^a ^1 and 5 represent the worst and best possible patient experiences respectively.

^b ^Items within these scales have five-point descriptive scales from "not at all" to "to a very large extent".

^c ^The three scales are scored from 0 to 100 where 100 represents the best possible experiences.

^d ^Items within the outcome scale have a five-point scale from "much worse" to "much better".

Factor analysis gave three factors accounting for 74.2% of the total variation (Table 2). Factor loadings were acceptable with all 15 items having loadings above 0.5 and loadings below 0.3 on the other two factors. In order of the total amount of variation that they explain, the factors can be described as relationship with health personnel, information and participation, and outcome. Table 2 shows that the item-total correlations and Cronbach's alpha for the final scales were acceptable, ranging from 0.65 to 0.89 and from 0.88 to 0.94, respectively.

Table 2 shows the mean scores for the final 15-item questionnaire on a scale of 0 to 100 where 100 is the best possible experiences of care. Scores were skewed towards positive experiences of care and ranged from 59.6 to 74.9 for the scales of information and participation and relationship with health personnel respectively.

The results of validity testing are shown in Table 3. The results of the 45 correlations were all highly significant. The three PEQ-CAMHS scores had moderate levels of correlation with overall satisfaction for how the child and parent were treated, the highest correlations being for the PEQ-CAMHS scale of relationship with health personnel. Slightly higher correlations were found for the child and parent outcomes, the highest being for the PEQ-CAMHS scores of outcome and relationship with health personnel. Moderate to high correlations were found for the two items relating to cooperation and the PEQ-CAMHS scores for relationship to health professional. Low to moderate correlations were found for the two items relating to parent involvement, the highest being for the PEQ-CAMHS scores for relationship with health personnel and information/participation. The item relating to information associated with side effects of medication had the highest correlation with PEQ-CAMHS scores for information and participation at 0.60. As expected the correlations for the variables relating to availability and waiting time had lower correlations, the highest being for PEQ-CAMHS scores of relationship with health personnel. Finally, correlations of a low level were found for responses to the two items relating to whether the health personnel had talked down to them or their child, the highest being for PEQ-CAMHS scores for relationship with health professional.

**Table 3:** Correlations between questionnaire scores and responses to individual questions

Variable	Relationship with health personnel	Information and participation	Outcome
Overall satisfaction^a^:			
Treatment for child	0.62	0.49	0.37
How parent was treated	0.64	0.47	0.28
Overall outcome^b^:			
For child	0.58	0.55	0.62
For parent	0.71	0.65	0.52
Cooperation^c^:			
Among health professionals at the clinic	0.73	0.65	0.40
Between the clinic and others involved with the child	0.62	0.56	0.30
Parent involvement:			
Parent understanding of treatment from the clinic^d^	0.51	0.56	0.33
Parent involvement in dialogue with the clinic^e^	0.26	0.24	0.09
Talked down to by personnel^c^:			
Child	0.25	0.15	0.11
Parent	0.41	0.25	0.15
Time and availability:			
Waiting time^f^	0.26	0.20	0.17
Acceptable number of hours with clinic^g^	0.45	0.35	0.26
Number of clinic visits in the last three months^h^	0.18	0.04	0.03
Contact with health professionals out of appointment time^c^	0.50	0.42	0.26
Information - side effects of medication^c^	0.47	0.60	0.37

All correlations are significant (p < 0.01).

^a ^Five-point scale: "very dissatisfied" to "very satisfied".

^b ^Five-point scale: "no benefit" to "a very large benefit".

^c ^Five-point scale: "Not at all" to "to a very large extent".

^d ^Five-point scale: "very poor" to "very good".

^e^Three-point scale: "never", "yes now and again", "yes often".

^f^Four-point scale: "no", "yes but not for long", "yes fairly long", "yes very long".

^g^Three-point scale: "very few hours", "too few hours", "enough hours".

^h^Four-point scale: "only once", "2-5 times", "6-12 times", "more than 12 times".

## Discussion

This study was part of the first national survey undertaken in Norway to assess parent satisfaction with all Norwegian outpatient CAHMS. The development of the PEQ-CAHMS Outpatients followed a review of the literature including existing questionnaires, interviews with parents, and consultation with an expert group of professionals involved in the delivery of CAMHS and researchers experienced in questionnaire and survey design. This was designed to ensure that the questionnaire covered the important aspects of outpatient care in sufficient detail and hence had content validity. Parents are important participants in the delivery of mental health care to their children [9] and are not only well placed to judge service quality but can also inform the process of development and hence the content of questionnaires that are designed to assess quality of care from their perspective. The resulting questionnaire underwent a rigorous process of piloting and testing for data quality, reliability and validity that have been recommended for evaluating such questionnaires [23].

The 15 items within the PEQ-CAMHS Outpatients contribute to three scales of relationship with health personnel, information and participation, and outcome. The levels of missing data in the range 1.5 to 5.6% suggest that it is acceptable to parents.

The results of factor analysis and tests of internal consistency were strong empirical support for the three scales. The questionnaire has evidence for construct validity following the application of hypotheses based on previous research findings and theory. In this national survey the questionnaire scores were approximately normally distributed in the range of 59.6 to 74.9 which means that there is potential for improvement in parent experiences over time and particularly for the scale of information and participation.

The test-retest reliability of the questionnaire was not assessed due to limited resources. Previous surveys undertaken in Norway have found acceptable levels of test-retest reliability for other questionnaires that assess user experiences [21,27]. Test-retest reliability will be considered for a subsample of parents taking part in a future national survey of user experiences of outpatient CAMHS in Norway. The tests of construct validity were based on other questionnaire items completed by the parents and hence did not include objective or clinical data. Further tests of construct validity should include comparisons with administrative and clinical variables available from the clinics.

The 15 items forming the three scales can be used alone if brevity is an important issue. Having just 15 items makes it more suitable for inclusion in a longer questionnaire alongside other instruments that are designed to assess the outcomes of care for example within a randomised controlled trial and other forms of evaluative study. The additional items included within the questionnaire that related to other aspects of care including information on medication and cooperation between health professionals, should also be considered for inclusion in future surveys. Some of these items were not relevant to all parents but were considered important enough for inclusion in the postal survey by the expert group and hence have content validity.

The questionnaire is being used in national surveys of parent experiences that have included 86 outpatient clinics across Norway and the results for the 2006 survey have been reported [22]. The results are included in the Norwegian national system of quality indicators and are designed to inform parent choice and quality improvement. They are available to parents and clinics in electronic and report form [22].

The questionnaire is designed for use with parents of children attending CAMHS outpatient clinics and while aspects of care that it assesses are of general relevance to CAMHS including the relationship with health personnel, it does not include aspects of inpatient care provision. The development of a questionnaire that included all aspects of CAMHS care would have necessitated a larger study that also included parents whose children had received inpatient care, both as a basis for informing the content of the questionnaire and also in testing. Some important aspects of parent experiences may be relevant for both inpatient and outpatient care and hence a generic questionnaire may be feasible. However, outpatient- and inpatient-specific modules will have greater content validity from a user perspective and are necessary for a more detailed understanding of the quality of CAMHS care. Furthermore, the PEQ-CAMHS Outpatients is designed for parent completion and hence does not include the views of children and adolescents. Future research should also seek to include the views of children and compare these with parent experiences and clinical data including outcomes.

The response rate of 46% lies within the range of 8 to 63% found following a review of surveys of parent satisfaction with child and adolescent mental health; the median response rate to postal surveys was 33% [17]. More recent postal surveys lie in the range 22% to 52% for a survey without reminders [16] and with a telephone reminder [9], respectively. 'Personal contact' or more direct approaches which include surveying parents as part of initial and follow-up procedures at CAMHS produce higher response rates [17] but there is greater potential for social desirability bias [32,33]. Parents may report higher levels of satisfaction because they feel that this will be more acceptable to those administering the survey. Non-response causes bias if non-respondents systematically differ from respondents in relation to important study variables, here parent experiences and satisfaction.

The findings for other follow-up surveys of Norwegian user experiences have shown that the response rates have not caused serious bias [33-36]. The response rate for the telephone survey was 56% following three postal contacts. There was no difference in overall parent experiences for respondents and telephone interviewees as assessed by a summary score comprising four items. However, there was one small but significant difference for one of these four items relating to "viewpoints being taken seriously". There was also a difference in the number of previous contacts with the clinic and education levels of parents. Interviewees reported a greater number of previous contacts and a smaller proportion had received higher education. The former may imply that non-respondents are parents of children with more severe conditions but this requires further investigation.

Parent satisfaction questionnaires have been criticised for lacking evidence relating to quantitative aspects of psychometric testing including reliability and validity [1,9,17]. The evaluation of the PEQ-CAMHS included assessing data quality, factor analysis to assess dimensionality, internal consistency and construct validity that have been recommended in the development of questionnaires designed to measure user experiences and satisfaction [23]. Few studies have undertaken such a comprehensive evaluation which followed consideration of content validity; that the questionnaire includes the important aspects of parent experiences with outpatient CAMHS. Moreover data quality, an important indicator of the acceptability of items, has rarely been reported in previous studies.

## Conclusions

The PEQ-CAMHS Outpatients includes important aspects of parent experiences with CAMHS that are based on the views of parents. The questionnaire has evidence for data quality, internal consistency, content validity and construct validity. The PEQ-CAMHS Outpatients is recommended for future applications designed to assess parent experiences of outpatient CAMHS and is being used in national surveys of parent experiences in Norway as a quality indicator. Test-retest reliability and further tests of construct validity, including comparisons with clinical variables are recommended for future research.

## Competing interests

The authors declare that they have no competing interests.

## Authors' contributions

All authors contributed to the design of the questionnaire and survey. AMG conducted the analysis and drafted the manuscript. OAB and OH were involved in data acquisition. All authors have made significant contributions by critically reviewing the manuscript and have read and approved the final version.
